# Colonic metastasis from breast carcinoma: a case report

**DOI:** 10.1186/s12957-017-1193-5

**Published:** 2017-07-05

**Authors:** Kazuma Tsujimura, Tsuyoshi Teruya, Masaya Kiyuna, Kuniki Higa, Junko Higa, Kouji Iha, Kiyoshi Chinen, Masaya Asato, Yasukatsu Takushi, Morihito Ota, Eijirou Dakeshita, Atsushi Nakachi, Akira Gakiya, Hiroshi Shiroma

**Affiliations:** 1 Department of Surgery, Tomishiro Central Hospital, 25 Azaueda, Tomishiro-shi, Okinawa 901-0243 Japan; 2 Department of Pathology, Tomishiro Central Hospital, Okinawa, Japan; 3 Department of Surgery, Nanbu Hospital, Okinawa, Japan

**Keywords:** Breast carcinoma, Colonic metastasis, Positron emission tomography-computed tomography

## Abstract

**Background:**

Colonic metastasis from breast carcinoma is very rare. Here, we report a case of colonic metastasis from breast carcinoma.

**Case presentation:**

The patient was a 51-year-old woman. She had upper abdominal pain, vomiting, and diarrhea, repeatedly. We performed abdominal contrast-enhanced computed tomography (CT) to investigate these symptoms. The CT scan revealed a tumor in the ascending colon with contrast enhancement and showed an expanded small intestine. For further investigation of this tumor, we performed whole positron emission tomography-computed tomography (PET-CT). The PET-CT scan revealed fluorodeoxyglucose uptake in the ascending colon, mesentery, left breast, and left axillary region. Analysis of biopsy samples obtained during colonoscopy revealed signet ring cell-like carcinoma. Moreover, biopsy of the breast tumor revealed invasive lobular carcinoma. Therefore, the preoperative diagnosis was colonic metastasis from breast carcinoma. Open ileocecal resection was performed. The final diagnosis was multiple metastatic breast carcinomas, and the TNM classification was T2N1M1 Stage IV.

**Conclusions:**

We presented a rare case of colonic metastasis from breast carcinoma. PET-CT may be useful in the diagnosis of metastatic breast cancer. When analysis of biopsy samples obtained during colonoscopy reveals signet ring cell-like carcinoma, the possibility of breast cancer as the primary tumor should be considered.

## Background

The primary areas of metastasis from breast carcinoma are the bones, lungs, pleura, liver, and brain. Gastrointestinal (GI) tract metastasis from breast carcinoma is relatively rare, and colonic metastasis from breast carcinoma is very rare [1, 2]. Here, we report a case of colonic metastasis from breast carcinoma.

## Case presentation

The patient was a 51-year-old woman without a relevant medical history. She presented at another hospital because of upper abdominal pain, vomiting, and diarrhea in July 2015. She was diagnosed with acute enteritis. Subsequently, she repeatedly experienced the same symptoms, and therefore, she underwent colonoscopy twice at this hospital. Colonoscopy indicated a bulge and mucosal inflammatory changes at the ileocecum. Biopsy of the lesion was performed twice; however, malignancy was not noted. Moreover, she was diagnosed with cholelithiasis, and she underwent laparoscopic cholecystectomy in November 2015. During the operation, white spots were noted at the greater omentum, abdominal wall, small intestinal serosa, and mesentery as intraperitoneal findings, and biopsies of the white spots at the greater omentum and abdominal wall did not indicate malignancy.

**Fig. 1:**
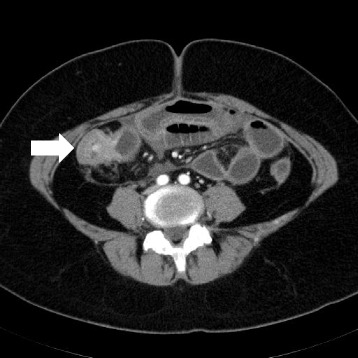
Contrast-enhanced computed tomography (CT). A contrast-enhanced CT image reveals a tumor with contrast enhancement and shows an expanded small intestine

**Fig. 2:**
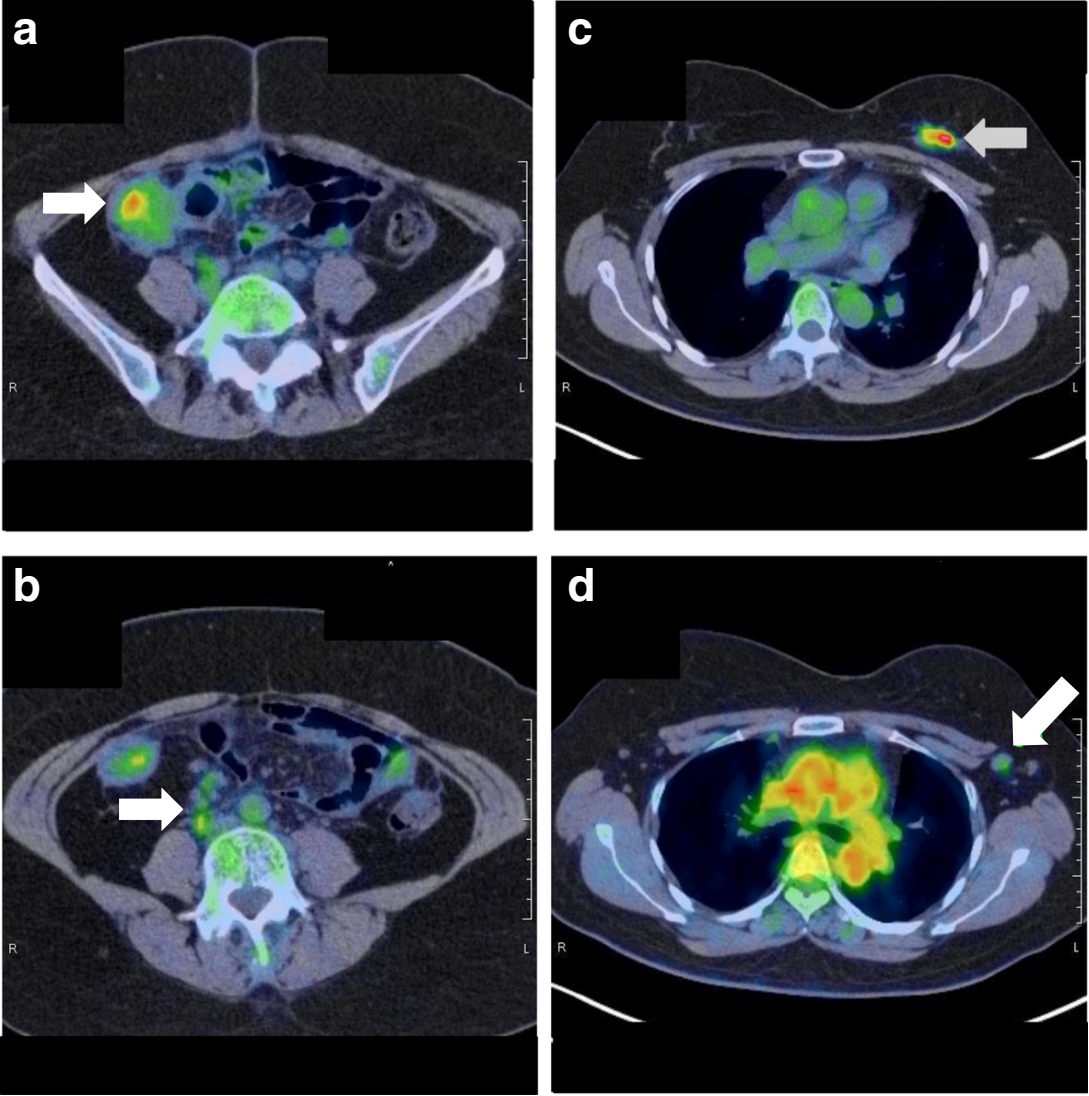
Positron emission tomography-computed tomography (PET-CT). **a** A whole PET-CT image shows fluorodeoxyglucose (FDG) uptake in the ascending colon. The maximum standardized uptake value (SUVmax) is 5.0. **b** A whole PET-CT image shows FDG uptake in the mesentery. The SUVmax is 2.8. **c** A whole PET-CT image shows FDG uptake in the left breast. The SUVmax is 6.4. **d** A whole PET-CT image shows FDG uptake in the left axillary region. The SUVmax is 2.1

**Fig. 3:**
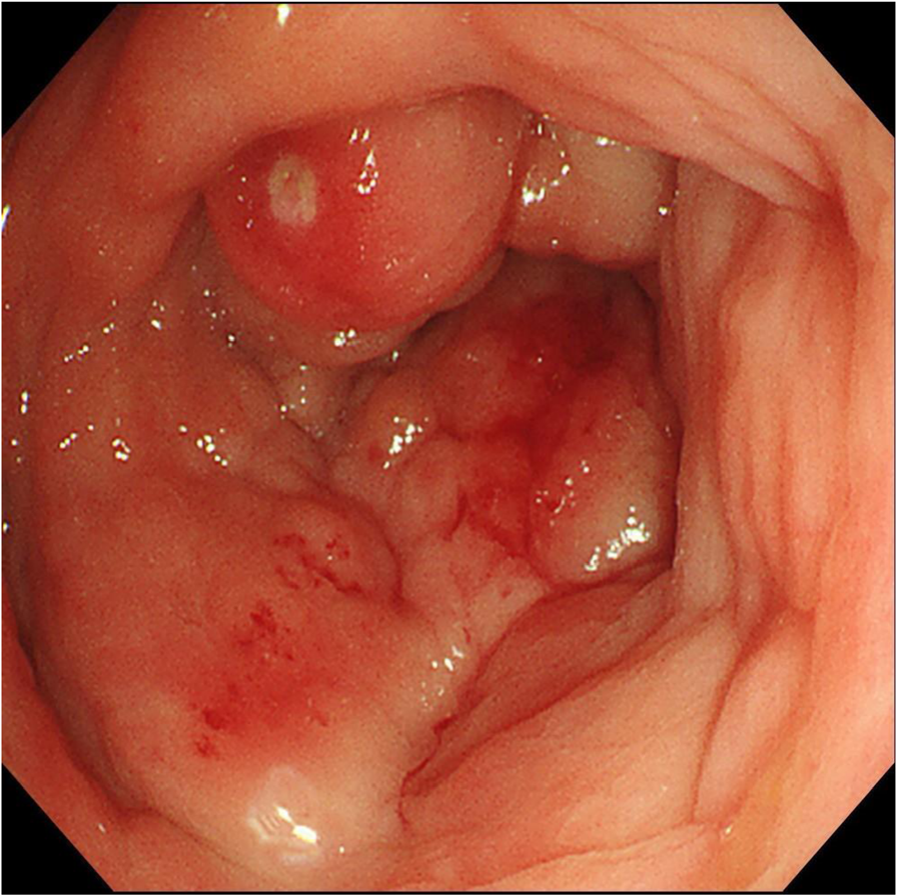
Colonoscopy. Colonoscopy shows swelling and stenosis of the ascending colon

**Fig. 4:**
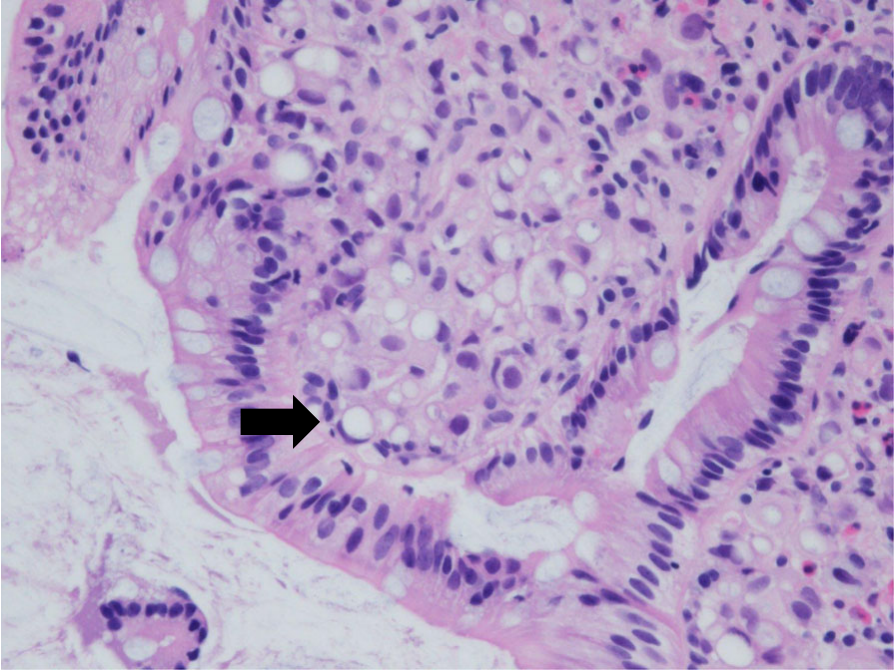
Hematoxylin and eosin (HE) staining. HE staining of the biopsy samples obtained during colonoscopy reveals signet ring cell-like carcinoma

**Fig. 5:**
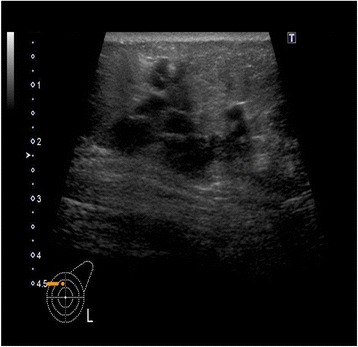
Ultrasonography (US). A US image of the breast shows an ill-defined, irregular, and hypervascular hypoechoic tumor measuring 36 × 22 × 22 mm

**Fig. 6:**
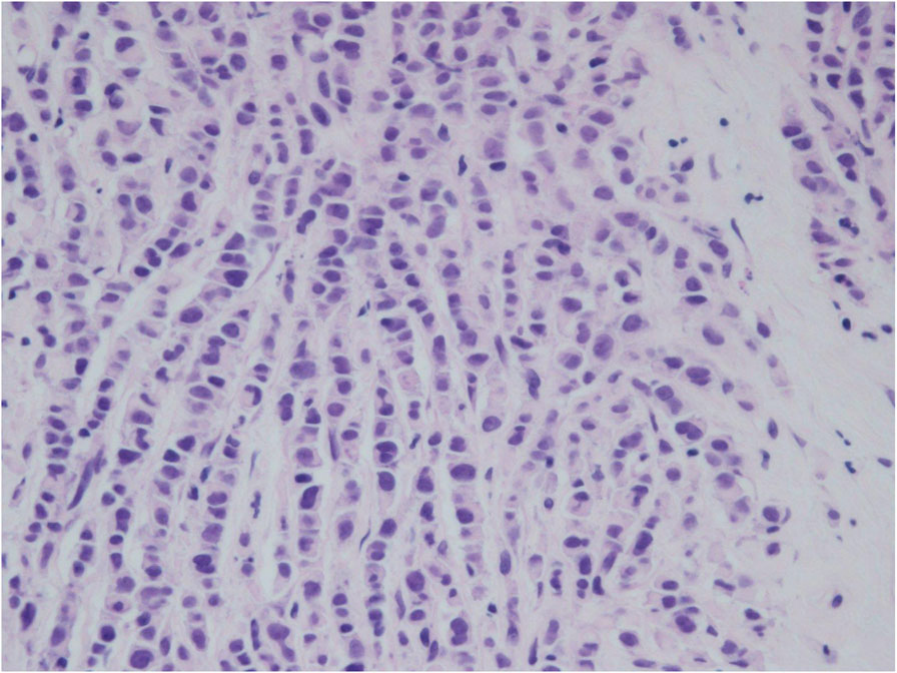
Hematoxylin and eosin (HE) staining. HE staining of the biopsy sample of the breast tumor reveals invasive lobular carcinoma

She again had the same symptoms in January 2016, and she was admitted to the same hospital for further examination and treatment. Eventually, for close examination, she was transferred to our hospital. In our hospital, abdominal contrast-enhanced computed tomography (CT) was performed to investigate her symptoms. The CT scan revealed a tumor in the ascending colon with contrast enhancement and showed an expanded small intestine (Fig. 1). For further investigation, we performed whole positron emission tomography-computed tomography (PET-CT) using Siemens True Point Biograph 16 (Siemens, Erlangen, Germany). The PET-CT scan revealed fluorodeoxyglucose (FDG) uptake in the ascending colon, mesentery, left breast, and left axillary region, with maximum standardized uptake values (SUVmax) of 5.0, 2.8, 6.4, and 2.1, respectively (Fig. 2a–d). Additionally, colonoscopy indicated swelling and stenosis of the ascending colon (Fig. 3), and we could not pass the endoscope to the ileocecum. Biopsies of the swelling and stenosis of the ascending colon were performed. Hematoxylin and eosin (HE) staining of the biopsy samples revealed signet ring cell-like carcinoma (Fig. 4). Upper gastrointestinal endoscopic examination showed normal findings. Moreover, we performed ultrasonography (US) and biopsy of the left breast tumor identified using PET-CT. US revealed an ill-defined, irregular, and hypervascular hypoechoic tumor measuring 36 × 22 × 22 mm (Fig. 5). HE staining of the breast tumor sample revealed invasive lobular carcinoma, and cadherin staining was negative (Fig. 6). In March 2016, the patient continued to experience abdominal pain and vomiting. Abdominal contrast-enhanced CT was performed again, and it revealed continuous ileus due to an ileocecal mass; therefore, open ileocecal resection was scheduled. With regard to tumor markers, before operation, the CA125 level was high (141 U/ml), while the CEA (3.2 ng/ml), CA19-9 (11.4 U/ml), and AFP (<2.0 ng/ml) levels were normal. The preoperative diagnosis was colonic metastasis from breast carcinoma.

**Fig. 7:**
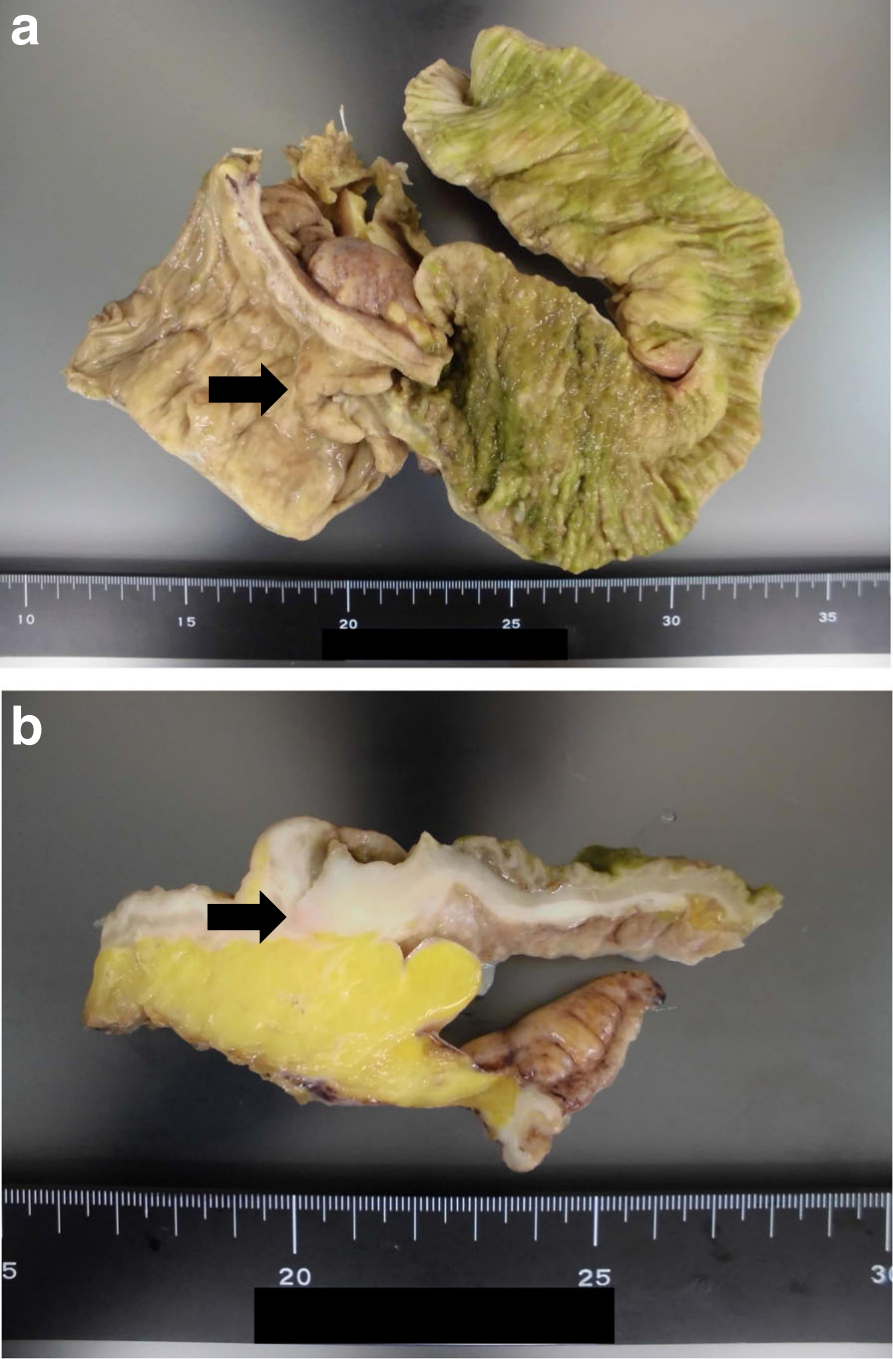
Surgical specimen. **a** A surgical ileocecal specimen shows a normal mucosal layer. The tumor involves the entire circumference of the intestinal tract and develops in an inward direction. The tumor size is 3 cm in the longitudinal direction. **b** A surgical ileocecal specimen shows a tumor in the submucosal region

**Fig. 8:**
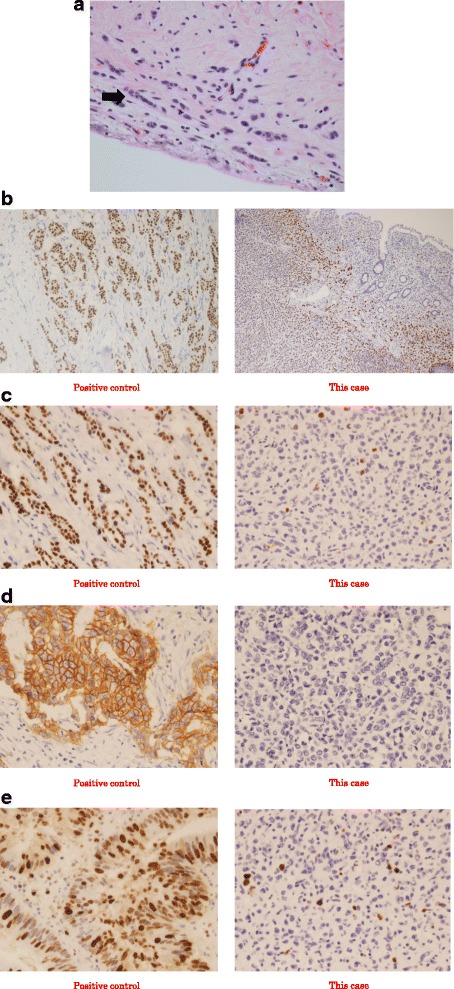
Histological examination. The findings of histological examination indicate invasive lobular carcinoma. **a** Hematoxylin and eosin staining of the tumor (×400). **b** Estrogen receptor (ER) staining of the tumor (×200). The positive rate of ER was 100%. **c** Progesterone receptor (PgR) staining of the tumor (×400). The positive rate of PgR was 2–3%. **d** Human epidermal growth factor receptor 2 (Her-2) staining of the tumor (×400). The positive rate of Her-2 was 0%. E Ki-67 staining of the tumor (×400). The positive rate of Ki-67 was 5–10%

Open ileocecal resection was performed, and white spots were noted at the peritoneum, pouch of Douglas, mesentery, and throughout the observed area. Biopsies of the spots were performed simultaneously. A surgical specimen from the ileocecal region showed a normal mucosal layer, and a tumor was noted at the submucosal region. Because the tumor involved the entire circumference of the intestinal tract and developed in an inward direction, there was stenosis of the ileocecum. The tumor size was 3 cm in the longitudinal direction (Fig. 7a, b). Histological examination revealed invasive lobular carcinoma (Fig. 8a). The positive rates of estrogen receptor, progesterone receptor, human epidermal growth factor receptor 2, and Ki-67 staining were almost 100, 2–3, 0, and 5–10%, respectively (Fig. 8b–e). The final diagnosis was multiple metastatic breast carcinomas, and the TNM classification was T2N1M1 stage IV. The histological grade of this tumor according to the College of American Pathologists (CAP) was grade 2 (score 7).

On postoperative day 8, anastomotic leakage occurred, and we performed open drainage and ileostomy. Subsequently, her general condition was good. We started treatment with letrozole. The duration of hospitalization was long, and she was discharged 1.5 months after open ileocecal resection. At 9 months after the operation, we continue to administer letrozole, and she has stable disease.

## Discussion

GI metastasis from breast carcinoma is relatively rare and has been reported to occur in 4–18% of disseminated breast cancer patients [3, 4]. Especially, the rate of colonic metastasis from breast carcinoma is extremely rare, with a frequency of approximately 3% in a review of the literature [5].

In the present case, there was continuous ileus due to an ileocecal mass, and open ileocecal resection was performed. Before the operation, PET-CT was useful for preoperative diagnosis, and the preoperative diagnosis was colonic metastasis from breast carcinoma. Generally, FDG-PET-CT is not recommended as the primary diagnostic procedure in breast cancer. Its sensitivity ranges from 48 to 96% and specificity from 73 to 100%. However, it has the potential to be useful for the detection of distant metastases. In such a case, its sensitivity has been reported to range from 80 to 100% and specificity from 75 to 100% [6]. In another report, for detecting distant metastatic disease, the sensitivity and specificity of FDG-PET-CT were shown to be greater than the sensitivity and specificity of conventional imaging [7]. However, FDG-PET-CT is not suitable for the detection of primary tumors owing to its low sensitivity for tumors measuring 0–10 mm [6]. In this case, we performed PET-CT to further investigate a tumor in the ascending colon that was identified using abdominal contrast-enhanced CT. As mentioned previously, PET-CT is not recommended as a primary diagnostic procedure in breast cancer. However, in this case, as distant metastasis was noted and the primary breast cancer was relatively large, we could make a preoperative diagnosis of colonic metastasis from breast carcinoma.

In this case, histological examination revealed invasive lobular carcinoma. Among the different histological types of breast carcinoma, it has been reported that lobular carcinoma and mucinous carcinoma metastasize relatively frequently to the GI tract [3, 8, 9]. One histological feature of colonic metastasis from breast carcinoma is the limited existence in the submucosa, muscles, and chorionic membrane layer. Especially, the tumor markedly invades the muscle layer, and the mucosal layer is normal [8, 10, 11]. As the mucosal layer is normal, it is essential to perform deep sampling during colonoscopy. In this case, biopsy during colonoscopy was performed thrice; however, a correct pathological diagnosis was not obtained. According to our findings, colonic metastasis from breast carcinoma is asymptomatic in the early stage, and symptoms due to stenosis occur only in the advanced stage, making early detection difficult. In this case, the tumor size was small in the longitudinal direction, but the tumor involved the entire circumference of the intestinal tract and developed in an inward direction. Therefore, there was continuous ileus due to stenosis of the ileocecum.

Invasive lobular carcinoma is associated with mucus in the intracellular region, and the histological features may indicate signet ring cell-like carcinoma [12]. When analysis of biopsy samples obtained during colonoscopy reveals signet ring cell-like carcinoma, colonic metastasis from breast carcinoma should be suspected. With regard to the treatment of colonic metastasis from breast carcinoma, even though temporizing treatment, narrow colon is resected and nutritional condition may be improved. Moreover, treatment success with chemotherapy and hormonotherapy after operation has been reported [13].

## Conclusions

PET-CT may be useful in the diagnosis of metastatic breast cancer. When analysis of biopsy samples obtained during colonoscopy reveals signet ring cell-like carcinoma, the possibility of breast cancer as the primary tumor should be considered.

## Abbreviations

CT: Computed tomography
ER: Estrogen receptor
FDG: Fluorodeoxyglucose
GI: Gastrointestinal
HE: Hematoxylin and eosin
Her-2: Human epidermal growth factor receptor 2
PET-CT: Positron emission tomography-computed tomography
PgR: Progesterone receptor
SUVmax: Maximum standardized uptake value
US: Ultrasonography.
